# Whole genome sequencing of coagulase positive staphylococci from a dog-and-owner screening survey

**DOI:** 10.1371/journal.pone.0245351

**Published:** 2021-01-11

**Authors:** Judit Sahin-Tóth, Eszter Kovács, Adrienn Tóthpál, János Juhász, Barbara Forró, Krisztián Bányai, Kata Havril, Andrea Horváth, Ágoston Ghidán, Orsolya Dobay

**Affiliations:** 1 Institute of Medical Microbiology, Semmelweis University, Budapest, Hungary; 2 Faculty of Information Technology and Bionics, Pázmány Péter Catholic University, Budapest, Hungary; 3 Institute for Veterinary Medical Research, Centre for Agricultural Research, Budapest, Hungary; Cornell University, UNITED STATES

## Abstract

**Background:**

*Staphylococcus aureus* and *S*. *pseudintermedius* are the two most common coagulase positive staphylococci (CPS). *S*. *aureus* is more prevalent among humans, whereas *S*. *pseudintermedius* is more commonly isolated from dogs, however, both can cause various community and hospital acquired diseases in humans.

**Methods:**

In the current study we screened 102 dogs and 84 owners in Hungary. We tested the antibiotic susceptibility of the strains and in order to get a better picture of the clonal relationship of the strains, we used pulsed-field gel electrophoresis. In addition, three pairs of isolates with identical PFGE patterns were whole genome sequenced, MLST and spa types were established.

**Results:**

Carriage rate of *S*. *aureus* was 23.8% in humans and 4.9% in dogs and two cases of co-carriage were found among dogs and owners. *S*. *pseudintermedius* carriage rate was 2.4% and 34.3%, respectively, with only one co-carriage. The isolates were generally rather susceptible to the tested antibiotics, but high tetracycline resistance of *S*. *pseudintermedius* strains was noted. The co-carried isolates shared almost the same resistance genes (including *tet(K)*, *bla(Z)*, *norA*, *mepR*, *lmrS*, *fosB*) and virulence gene pattern. Apart from the common staphylococcal enzymes and cytotoxins, we found enterotoxins and exfoliative toxins as well. The two *S*. *aureus* pairs belonged to ST45-t630, ST45-t671 and ST15-t084, ST15-t084, respectively. The co-carried *S*. *pseudintermedius* isolates shared the same housekeeping gene alleles determining a novel sequence type ST1685.

**Conclusions:**

Based on the genomic data, dog-owner co-carried strains displayed only insignificant differences therefore provided evidence for potential human-to-dog and dog-to-human transmission.

## 1. Introduction

*S*. *aureus* is an important pathogen causing a broad range of diseases including community acquired and nosocomial skin and soft tissue infections, and life threatening conditions such as bloodstream infections. Not only humans are affected but several different animal species as well. The human nose is the main niche of *S*. *aureus*, which can persistently colonize around 30% of the population. This non-fastidious bacterium can survive in the environment for longer periods and it has a zoonotic potential as well. Several articles provided evidence that animals can act as reservoirs for these bacteria, and numerous cases have been reported where humans were infected with animal related strains [1,2]. The emergence of methicillin resistant strains (MRSA) in the 1960s made the fight more difficult against this pathogen and raised significant concern about animal and public health [2,3]. MRSA strains were originally hospital-acquired but then genotypically different community-acquired MRSA strains emerged and finally in the early 2000s livestock associated MRSA strains were also detected. The most wide spread LA-MRSA clonal complex is CC398 which was originally found in conventionally raised pigs [4,5].

*S*. *pseudintermedius* is another coagulase-positive staphylococcus (CPS) that is a common veterinary pathogen mainly colonizing small animals such as dogs and cats [6]. In animals it is mainly responsible for secondary infections like surgical site infection, superficial and deep pyoderma [7]. In recent years, methicillin-resistant *S*. *pseudintermedius* (MRSP) have been identified from clinical samples of canine origin. The PFGE analysis of these strains revealed that the owners often carried the same strains [8–10]. These results raised questions of interspecies pathogen transmission and resistance gene transfer between bacterial species [11]. In recent years, human skin and medical device associated bloodstream infections, food poisoning cases have also been occasionally reported [6,12–16] raising more attention to this species in human healthcare.

Our aim was to assess CPS prevalence in dogs and their owners in Hungary and compare human and animal strains.

## 2. Materials and methods

### 2.1 Phenotypic and genotypic identification of the CPS isolates

For the presence of CPS, we screened 102 dogs and their respective 84 owners in Budapest (60.7%) and 14 other towns (39.3%) in Hungary. Healthy dog owners and their animals were recruited for this research, no laboratory animals were used during this survey. No additional information was collected about the participants. Dog samples were collected from the nose, mouth and skin of the head with three different swabs, whereas a single nasal specimen from both nostrils was taken from the owners. All swabs were transported to the laboratory in Amies transport media (Transwab, Medical Wire & Equipment, Corsham, UK).

Swab samples were inoculated onto blood agar plates and incubated overnight at 37°C in 5% CO_2_. CPS were identified by colony morphology (ß-hemolytic colonies with golden or porcelain white pigment production) and biochemical tests: catalase and clumping test (Pastorex Staph-Plus Kit, Bio-Rad, Marnes-la-Coquette, France).

MALDI-TOF and PCR based techniques were used to confirm the assumptive phenotypic identification. Putative *S*. *aureus* isolates were confirmed by an in-house *nucA-mecA* duplex PCR [17]. To differentiate *S*. *intermedius* and *S*. *pseudintermedius*, *pta* PCR-RFLP was used: the *MboI* digestion of the *pta* PCR product of *S*. *pseudintermedius* resulted in two fragments, whereas the *pta* gene of *S*. *intermedius* does not have a *MboI* recognition site [18].

Genetic relatedness of the strains was determined by pulsed-field gel electrophoresis [19]. The gel pictures were analysed by the Fingerptinting II software (Bio-Rad, Marnes-la-Coquette, France). The spa type of the *S*. *aureus* isolates was determined by Sanger sequencing at the Biomi Ltd., Gödöllő, Hungary.

The number of ethical permit issued by the Semmelweis University Regional and Institutional Committee of Science and Research Ethics is: SE RKEB 181/2020. The specimens were collected non-invasively from both humans and dogs, with a soft cotton swab from the mucosal surface or skin.

### 2.2. Antibiotic susceptibility of the CPS isolates

Antibiotic sensitivity to penicillin, oxacillin, erythromycin, clindamycin, tetracycline, gentamicin, ciprofloxacin, mupirocin was determined by agar dilution, while disc diffusion was used in case of cefoxitin applying the EUCAST breakpoints [20]. Inducible resistance to clindamycin was performed by D-test where it was necessary.

### 2.3. Genome sequencing and data analysis

Whole genome sequencing of four CPS isolates originating from the dog-owner co-carriage cases (Q37, Q38, Q81, Q82), as well as two CPS isolates sharing the same PFGE pattern (Q85, Q47), was performed.

For DNA preparation, first an in-house method was used to lyse the cell wall of the bacteria. A bacterial suspension was made with 200 μl saline buffer and centrifuged at 8000 x g for two minutes. The supernatant was discarded and the cells were resuspended in the following lysis solution: 165 μl EC lysis buffer [1M NaCl, 100 mM EDTA, 0.5% Brij58, 0.2% deoxycholate, 0.5% N-lauroyl sarcosine], 15 μl lysostaphin (1mg/ml stock solution) and 20 μl lysozyme (20mg/ml stock solution) The mixture was incubated at 37°C for 30 minutes. Subsequently, the DNA was purified by the ZR Fungal/Bacterial DNA MiniPrep according to the manufacturer's instructions. (Zymo Research Corp., Irvine, CA, US).

DNA libraries compatible with Illumina sequencing was carried out using the Nextera XT kit and Nextera XT Index Kit v2 Set A (Illumina, San Diego, CA, USA). Whole genome sequencing was performed on NextSeq 500 Illumina equipment (Illumina, Inc. San Diego, CA USA) using NextSeq 500/550 Mid-Output Kit, resulting in 2x75 bp long paired end reads.

De novo assembly was performed by SPAdes (http://cab.spbu.ru/software/spades/) [21], annotation by RAST (https://rast.nmpdr.org/) [22] and MAUVE (http://darlinglab.org/mauve/mauve.html) [23] was used to rearrange and align the annotated scaffolds. Detection of resistance genes was carried out with ResFinder 2.3 (https://cge.cbs.dtu.dk/services/ResFinder/) [24] (with the default settings) and CARD (https://card.mcmaster.ca/) [25]. For MLST typing MLST 2.0 was used (https://cge.cbs.dtu.dk/services/MLST/) [26]. The allelic profiles of the isolates were compared with allele sequences present in the PubMLST database (https://pubmlst.org/) [27,28]. Virulence and toxin genes were detected by VirulenceFinder 2.0 (https://cge.cbs.dtu.dk/services/VirulenceFinder/) [29] (with the default settings) and by manual search based on the Virulence Factors for Pathogenic Bacteria database (http://www.mgc.ac.cn/VFs/) [30]. The identified genes and proteins were compared by using Clustal Omega Multiple Sequence Alignment (https://www.ebi.ac.uk/Tools/msa/clustalo/) [31].

The genome sequences have been uploaded to NCBI GenBank, under the following accession numbers: Q37: JAEDAR000000000, Q38: JAEDAS000000000, Q47: JAEDAT000000000, Q85: JAEDAU000000000, Q81: JAEDAV000000000, Q82: JAEEAO000000000.

## 3. Results

### 3.1. Carriage rate and sample distribution

*S*. *aureus* carriage rate was 23.8% (20/84) among humans and 4.9% (5/102) in dogs. We found two cases of *S*. *aureus* positivity in both dogs and their owners (samples: Q37-Q38, Q84-Q85). *S*. *pseudintermedius* carriage showed an inverse pattern: 2.4% (2/84) prevalence in humans and 34.3% (35/102) prevalence in dogs and we had only one co-carriage case (sample Q81 and Q82).

**Fig 1:**
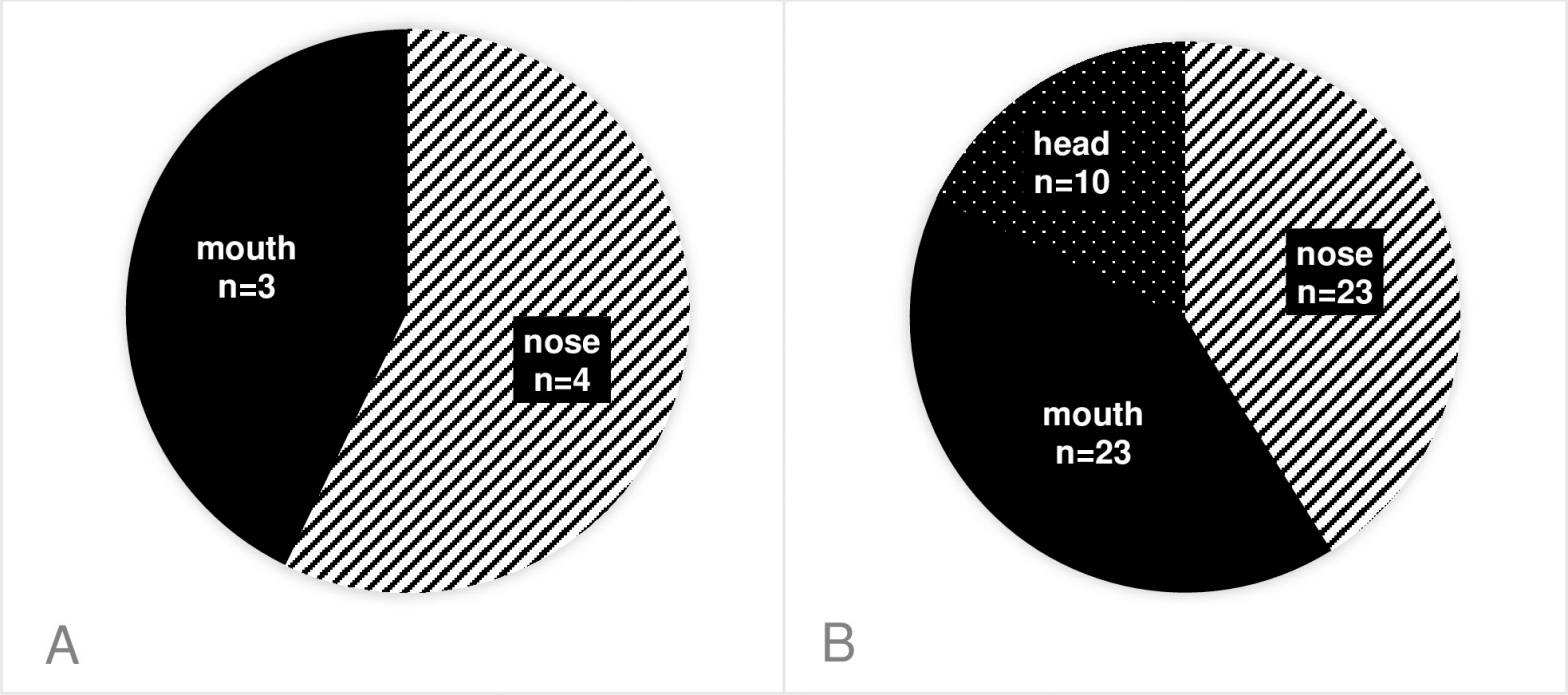
Distribution of the staphylococcal isolates among different sampling sites in dogs. (A): *S*. *aureus*, (B): *S*. *pseudintermedius*.

Altogether 27 *S*. *aureus* and 58 *S*. *pseudintermedius* strains were isolated, and *S*. *intermedius* was not found. In dogs, we could isolate the CPSs from the following body sites: *S*. *pseudintermedius* isolates mainly from the nose (23/56) and the mouth (23/56), the rest were found on the head (10/56). *S*. *aureus* was not isolated from the head at all, only from the nose (4/7) and the mouth (3/7). (Fig 1) Although the same bacteria could be isolated from multiple anatomical sites of the same canine hosts in 18 cases, but only one specimen per host was enrolled in the study. Hence the final number of isolates involved in further investigations was n = 25 *S*. *aureus* and n = 37 *S*. *pseudintermedius*.

### 3.2. Antibiotic susceptibility results

We found that 60% of the *S*. *aureus* strains showed resistance to penicillin, but all of them were sensitive to cefoxitin and oxacillin. Furthermore all of the *S*. *aureus* isolates were sensitive to mupirocin, ciprofloxacin and gentamicin. Only 8% were resistant to tetracycline, 4% to clindamycin and erythromycin and one isolate was multiresistant (i.e., resistant to at least three different antibiotic classes).

Surprisingly only 32% of the *S*. *pseudintermedius* isolates were resistant to penicillin, on the other hand 57% of them showed resistance to tetracycline, 14% to erythromycin, 11% to clindamycin and gentamicin each and 3% to ciprofloxacin. Seven isolates were multiresistant. All of them were sensitive to the locally administrable mupirocin, and to oxacillin and cefoxitin (Table 1).

**Table 1:** Antibiotic susceptibility results of the CPS isolates.

Antibiotic	Species	MIC range (mg/l)	Sensitive (%)	Intermediate (%)	Resistant (%)
PEN	*S*. *aureus*	0.06–32	60.0	0.0	40.0
	*S*. *pseudintermedius*	0.06–32	67.6	0.0	32.4
OXA	*S*. *aureus*	0.125–0.5	100.0	0.0	0.0
	*S*. *pseudintermedius*	0.06–0.125	100.0	0.0	0.0
MUP	*S*. *aureus*	<0.06	100.0	0.0	0.0
	*S*. *pseudintermedius*	<0.06	100.0	0.0	0.0
CLI	*S*. *aureus*	0.06–0.25	96.0	0.0	4.0
	*S*. *pseudintermedius*	0.094->256	89.2	0.0	10.8
ERY	*S*. *aureus*	0.047->128	96.0	0.0	4.0
	*S*. *pseudintermedius*	0.125->128	86.0	0.0	14.0
TET	*S*. *aureus*	0.25–64	92.0	0.0	8.0
	*S*. *pseudintermedius*	0.06–64	43.2	0.0	56.8
CIP	*S*. *aureus*	0.25–1	100.0	0.0	0.0
	*S*. *pseudintermedius*	0.19->32	97.3	0.0	2.7
GEN	*S*. *aureus*	0.125–1	100.0	0.0	0.0
	*S*. *pseudintermedius*	0.125->2	89.2	0.0	10.8
FOX	*S*. *aureus*	26-29mm	100.0	0.0	0.0
	*S*. *pseudintermedius*	36-41mm	100.0	0.0	0.0

PEN = penicillin, OXA = oxacillin, MUP = mupirocin, CLI = cindamycin, ERY = erythromycin, TET = tetracycline, CIP = ciprofloxacin, GEN = gentamicin, FOX = cefoxitin.

All four co-carried *S*. *aureus* isolates were penicillin resistant, moreover the Q37-Q38 isolates showed elevated minimal inhibitory concentrations to tetracycline (Table 2).

**Table 2:** Antibiotic susceptibility of the co-carried CPS isolates.

Sample	Origin	Secies	PEN		OXA		FOX		MUP		CLI		ERY		TET		CIP		GEN	
Q37	human	AUR	16	R	0.5	S	29mm	S	<0.06	S	0.06	S	0.125	S	64	R	0.5	S	0.125	S
Q38	dog	AUR	32	R	0.25	S	26mm	S	<0.06	S	0.125	S	0.125	S	64	R	0.5	S	0.125	S
Q47	human	AUR	8	R	0.25	S	28mm	S	<0.06	S	0.125	S	0.19	S	0.25	S	0.5	S	0.25	S
Q85	dog	AUR	2	R	0.125	S	27mm	S	<0.06	S	0.06	S	0.125	S	0.25	S	0.5	S	0.125	S
Q81	human	PSE	<0.06	S	0.125	S	39mm	S	<0.06	S	0.125	S	0.064	S	0.25	S	0.5	S	0.5	S
Q82	dog	PSE	<0.06	S	0.125	S	40mm	S	<0.06	S	0.125	S	0.047	S	0.25	S	0.5	S	0.5	S

AUR *= S*. *aureus*, PSE *= S*. *peudinermedius*; R = resistant, S = sensitive, PEN = penicillin, OXA = oxacillin, MUP = mupirocin, CLI = cindamycin, ERY = erythromycin, TET = tetracycline, CIP = ciprofloxacin, GEN = gentamicin, FOX = cefoxitin.

### 3.3. PFGE and spa typing results

**Fig 2:**
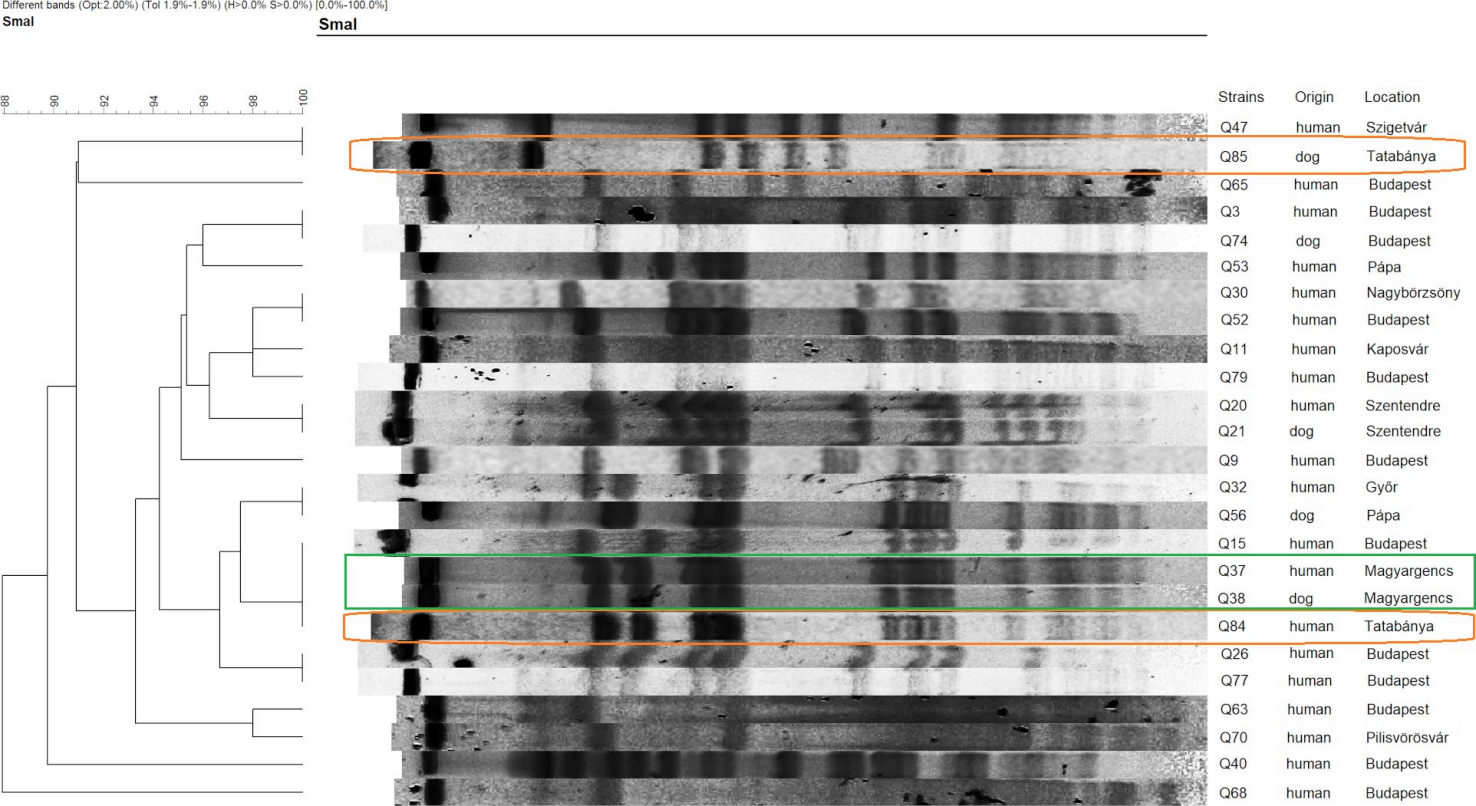
PFGE dendrogram of the *S*. *aureus* isolates. The co-carried strains, Q37-Q38 and Q84-85, are circled on the dendogram. Among these isolates Q37-Q38-Q84 showed the same banding pattern while Q85 shared the pattern of an isolate (Q47) from a diferrent location.

**Fig 3:**
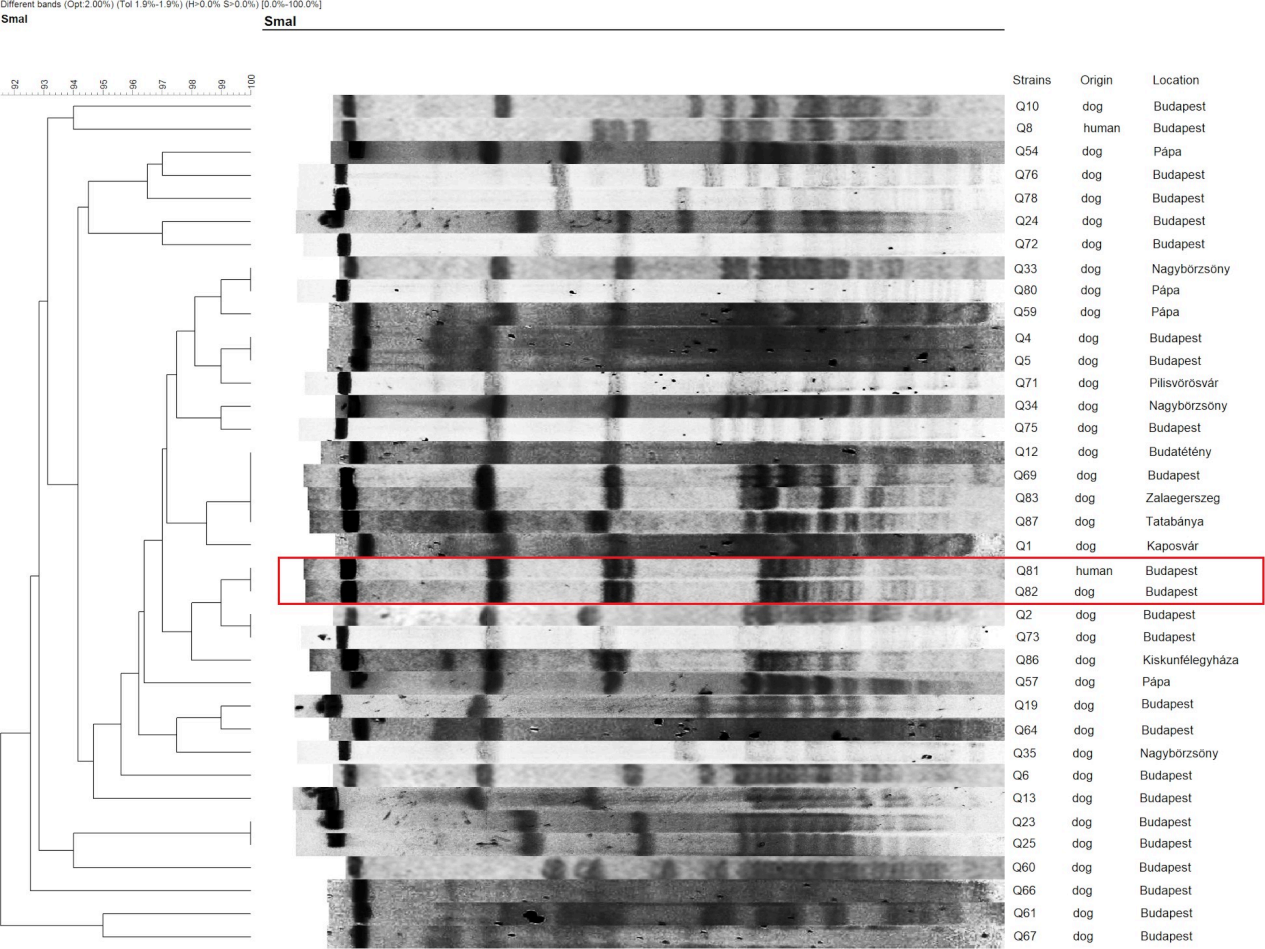
PFGE dendrogram of the *S*. *pseudintermedius* isolates. The co-carried strains indicated on the dendogram are Q81 and Q82.

The PFGE analysis of *S*. *aureus* (n = 25) showed the presence of isolates with similar patterns in different towns. Only one of the two carried pairs had the same banding pattern (Q37-Q38). The strains of the other pair (Q84-Q85) differed from one another, but the owner’s sample (Q84) shared the pattern of the previous pair, while the dog’s sample (Q85) was similar to a human *S*. *aureus* isolate (Q47) from a different town (Fig 2). Regarding *S*. *pseudintermedius* (n = 37), similarities were observed between strains from different places, and also from unrelated human-dog specimens. The only dog-owner co-carriage pair (Q81-Q82) had the same macrorestriction pattern (Fig 3).

Spa typing is a single-locus typing technique, hence it provides a more precise sub-typing compared to MLST and several spa types can be linked to a single clonal complex. Accordingly, the ST15 isolates (see below) both belonged to t084 spa type, while the ST45 isolates were classified into t630 and t671.

### 3.4. Whole genome sequencing results

We chose the co-carried strains with identical PFGE gel patterns for further analysis with whole genome sequencing (WGS): Q37 and Q38 (*S*. *aureus* isolates) and Q81-Q82 (*S*. *pseudintermedius* isolates). We were also interested in the genomic relationship between a canine origin *S*. *aureus* isolate (Q85) and a human isolate (Q47) with shared PFGE pattern but of different geographical origin.

**Fig 4:**
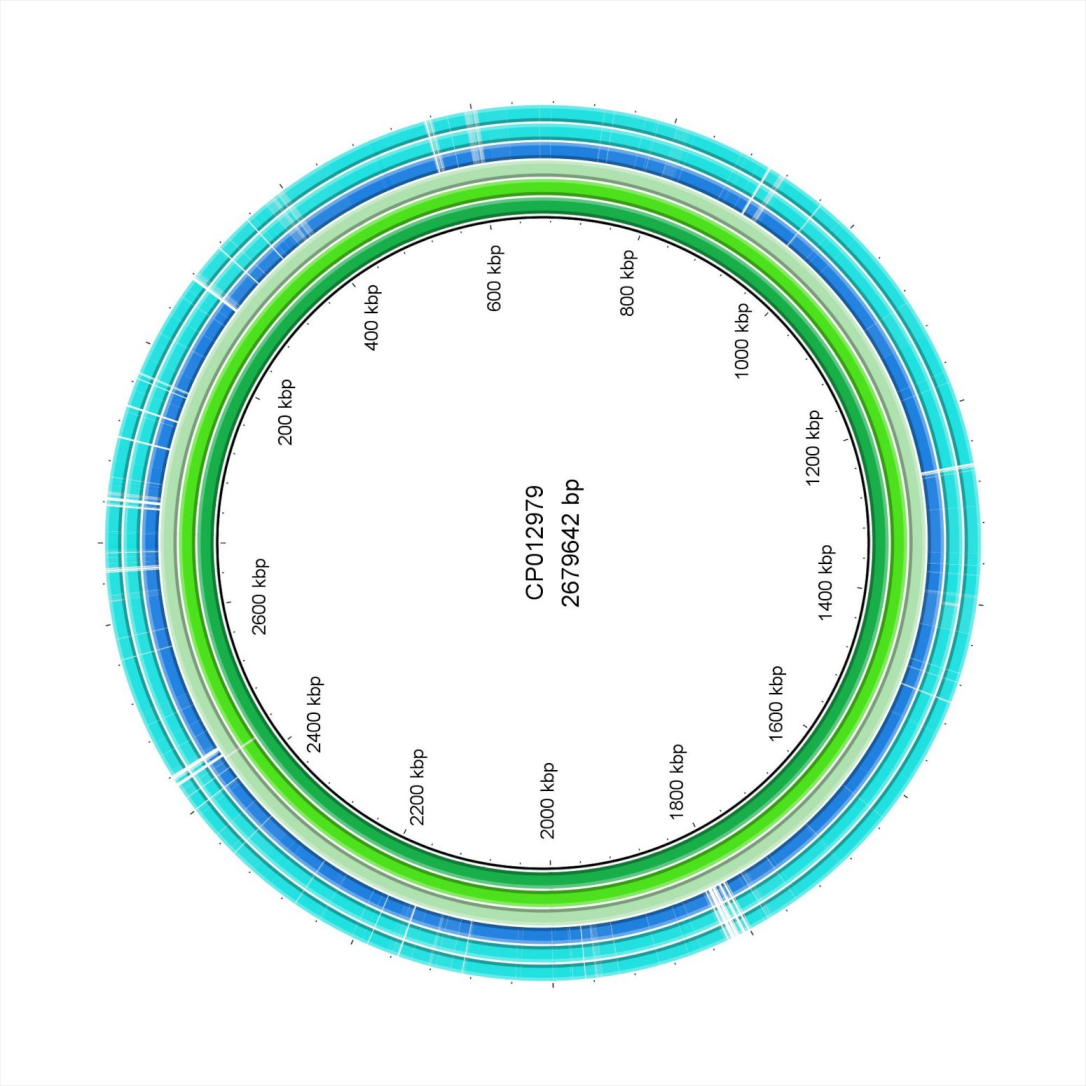
BRIG diagram of the *S*. *aureus* isolates. The order of isolates from inside out is the following: CP012979 reference strain, Q37, Q38, CP014791 reference strain, Q47, Q85.

**Fig 5:**
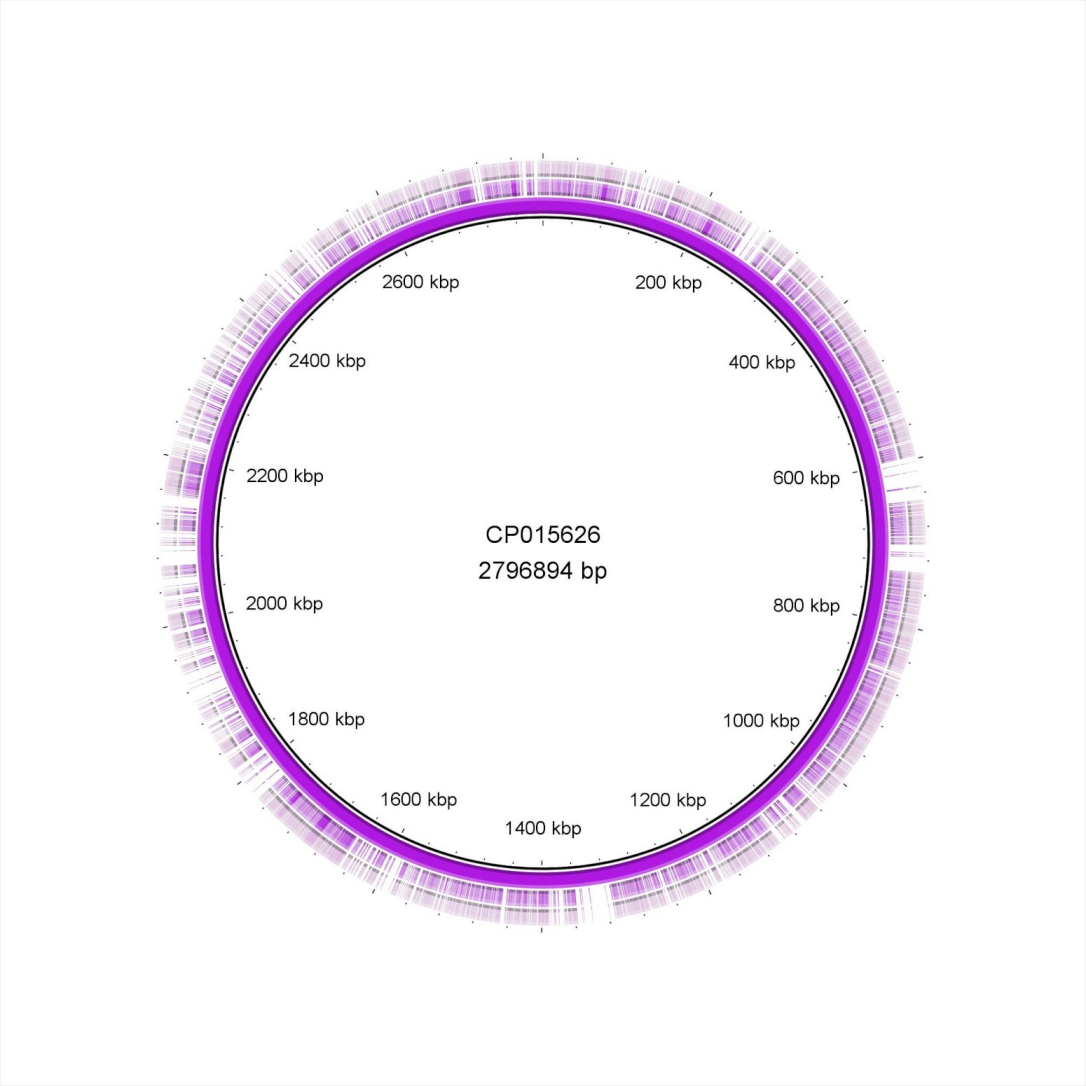
BRIG diagram of the *S*. *pseudintermedius* isolates. The order of isolates from inside out is the following: CP015626 reference strain, Q81, Q82.

The Brig diagrams showed that the Q37-38 and Q81-82 isolates were nearly identical, while minor differences were detected in the Q47 and Q85 samples (Figs 4 and 5). Detalied genomic analysis is provided below.

#### 3.4.1. MLST results

MLST results were extracted from the NGS data, based on a set of seven housekeeping genes commonly used in genotyping of staphylococci. The MLST analysis revealed that the Q37-Q38 isolates belonged to ST15, while Q47-Q85 were part of ST45. The *S*. *pseudintermedius* strains shared the same house-keeping gene profiles, but displayed a novel allele combination (10-24-4-2-19-26-2), which was assigned as ST1685 by the MLST curators (Table 3).

**Table 3:** Characterization of the co-carried isolates.

Isolate	Species	Origin	Sampling location	ST—spa typing	Resistance phenotype	Resistance genotype
Q37	AUR	human nasal swab	Magyargencs	ST15-t084	PEN,TET	*blaZ*, *tet(K)*, *norA*, *lmrS*, *mepR*, *fosB*
Q38	AUR	dog nasal swab	Magyargencs	ST15-t084	PEN, TET	*blaZ*, *tet(K)*, *norA*, *lmrS*, *mepR*
Q47	AUR	human nasal swab	Szigetvár	ST45-t630	PEN	*blaZ*, *norA*, *lmrS*, *mepR*
Q85	AUR	dog nasal swab	Tatabánya	ST45-t671	PEN	*blaZ*, *norA*, *lmrS*, *mepR*
Q81	PSE	human nasal swab	Budapest	ST1685	-	-
Q82	PSE	dog oral swab	Budapest	ST1685	-	-

AUR *= S*. *aureus*, PSE *= S*. *peudinermedius*; PEN = penicillin, TET = tetracycline.

#### 3.4.2. Resistance genes

All four *S*. *aureus* isolates carried the ß-lactamase gene *blaZ*; the multidrug efflux pump *norA* with its positive regulator *mgrA*, *arlS*, *arlR* which confers resistance to fluoroquinolones; *mepR* which is the upstream repressor of *mepA*, an efflux protein, the presence of which can lead to tetracycline and glycylcycline resistance; *lmrS* major facilitator superfamily antibiotic efflux pump which is capable of expelling a variety of antibiotics when expressed [25].

The Q37-Q38 pair also possessed the *tet(K)* gene responsible for tetracycline efflux and the *fosB* gene coding for a thiol transferase which can lead to fosfomycin resistance through the inactivation of the antibiotic [25].

In the Q47-85 pair, the I45M amino acid change was identified in the topoisomerase GrlA which could lead to elevated ciprofloxacin MIC. E291D, T396N amino acid changes were found in MurA transferase and A100V in GlpT transporter which all can confer fosfomycin resistance through either the overexpression of MurA or the reduced import of fosfomycin into the bacteria [24]. No resistance genes were found in the *S*. *pseudintermedius* isolates.

#### 3.4.3. Virulence genes: Biofilm production, adhesion factors, anti-opsonization factors

All four *S*. *aureus* isolates contained the ica operon which is responsible for the poly-n-succinyl-β-1,6 glucosamine (PNSG) polysaccharide production during infection allowing bacteria to adhere to each other thus promoting biofilm formation. In addition, several further genes were found responsible for the production of microbial surface components recognizing adhesive matrix molecules (MSCRAMMs) such as clumping factors (*clfA*, *clfB*), collagen binding protein (*cna*), extracellular adherence protein (*map*), elastin binding protein (*ebp*), fibronectin binding proteins (*fnbA*, *fnbB*) or Ser-Asp rich proteins (*sdrE*, *sdrC*). The protein products of these genes are also promoting biofilm formation but through different pathways than the ica operon. They can specifically bind to several different extracellular matrix molecules therefore they can promote the assembly of bacterial films [30]. Phenol-soluble modulins (*psmβ1;psmß2*) were also found which can facilitate biofilm development, dissemination of biofilm-associated infections in the human body and can play a role in the stimulation of inflammatory responses [32].

All our isolates carried different capsule genes promoting capsule production enabling the bacteria to avoid opsonization and subsequent phagocytosis. The staphylococcal immunoglobulin binding protein gene (*sbi*) was also detected.

The *S*. *pseudintermedius* isolates contained the ica operon and quorum sensing genes (*agrA-agrD*), and phenol-soluble modulin proteins (*psmß*). An aureus-like immunoglobulin binding protein gene (*sbi*) was also detected.

All genes found in the strains are summarised in Tables 4 and 5.

**Table 4:** Virulence genes in the co-carried *S*. *aureus* strains.

*S*. *aureus* strains		Q37	Q38	Q47	Q85
		owner	dog	owner	dog
leukocidins	*lukD*				
	*lukE*				
	*lukS-PV*				
	*lukF-PV*				
hemolysins	*hla*				
	*hlb*				
	*hld*				
	*hlg-a*				
	*hlg-b*				
	*hlg-c*				
tss toxin	*tsst*				
exfoliatve toxins	*eta*				
	*etb*				
enterotoxins	*sea*				
	*seb*				
	*sec*				
	*sed*				
	*see*				
	*seg*				
	*seh*				
	*sei*				
	*sej*				
	*sek*				
	*sel*				
	*sem*				
	*sen*				
	*seo*				
	*seu*				
ica operon	*icaA*				
	*icaB*				
	*icaC*				
	*icaD*				
	*icaR*				
adherence	*clfA*				
	*clfB*				
	*fnbA*				
	*fnbB*				
	*bap*				
	*cna*				
	*ebpS*				
	*sdrC*				
	*sdrD*				
	*sdrE*				
	*atl*				
	*map*				
	*psm-α*				
	*psm-ß1*				
	*psm-ß2*				
enzymes	*sspA*				
	*sspB*				
	*sspC*				
	*hysA*				
	*lip*				
	*geh*				
	*coa*				
	*sak*				
	*nuc*				
	*aur*				
	*vWbp*				
immune evasion	*spa*				
	*adsA*				
	*cap*				
	*chp*				
	*sbi*				
	*scn*				

Gray shading: Positivity, no shading: Negativity.

**Table 5:** Virulence genes in the co-carried *S*. *pseudintermedius* strains.

*S*. *pseudintermedius* strains		Q81	Q82
		owner	dog
leukocidins	*lukS-I*		
	*lukF-I*		
hemolysins	*hla*		
	*hlb*		
	*hld*		
	*hlg-a*		
	*hlg-b*		
	*hlg-c*		
	*hly-III*		
exfoliative toxins	*expA*		
	*expB*		
	*siet*		
enterotoxins	*sea*		
	*seb*		
	*sec*		
	*sed*		
	*see*		
	*sec-int*		
adherence	*icaA*		
	*icaB*		
	*icaC*		
	*icaD*		
	*icaR*		
	*agrA*		
	*agrB*		
	*agrC*		
	*agrD*		
	*psm-α*		
	*psm-ß*		
enzymes	*coa*		
	*nuc*		
immune evasion	*sbi*		

Gray shading: Positivity, no shading: Negativity.

#### 3.4.4. Virulence genes: Enzymes, toxins

*S*. *aureus* isolates possessed enzymes contributing to tissue invasion and destruction [33]: aureolysin (*aur*), hyaluronate lysate (*hysA*), lipase (either *geh* or *lip* or both), serine protease (*sspA*), staphopain (*sspB*, *sspC*), staphylocoagulase (*coa*), von Willebrand factor binding-protein (*vWbp*), staphylococcal thermonuclease (*nuc*).

All of the isolates carried hemolysins: *hla*, *hlb*, *hld*, *hlg-a*, *hlg-b; hlg-c*. However only the Q37-Q38 pair owned leukocidins like *lukE* and *lukD*. With the help of these cytotoxins the bacterium is able to lyse host cell membranes [33]. Interestingly both Q47 and Q85 possessed several staphylococcal enterotoxin (SE) genes: *seg*, *sei*, *sem*, *sen*, *seo*, *seu* were found in both, but Q47 had *sec* and *sel* as well. These are heat-stable toxins which are associated with staphylococcal food poisoning [33].

*S*. *pseudintermedius* samples contained the constitutive enzyme genes like coagulase (*coa*) and thermonuclease (*nuc*). From the cytotoxin coding genes *hlb*, *hly-III* hemolysins and *lukS* leukocidin were carried by the strains. Both of them were enterotoxin (*sec-int*) and exfoliative toxin (*siet*, *expB*) positive as seen in Table 5.

## 4. Discussion

### 4.1.Carriage

*S*. *aureus* nasal carriage in the general human population is around 20–30% [34–36]. In our previous human carriage studies, the nasal colonisation rates were found to be 29.3% among university students [17] and 21.3% among children attending day-care centres [37]. The 23.8% carriage rate observed in this study is in good correlation with other findings. Literature data on *S*. *aureus* colonization of healthy dogs in community environments is very limited. According to different sources it varies between 2–8% [6,38,39]. Our results seem to further support these numbers: we found 4.9% prevalence in dogs. *S*. *aureus* was more commonly present in the nares and mouth rather than the skin of a companion dog. Therefore these sites should be the primary sampling targets when a study is designed.

*S*. *pseudintermedius* nasal carriage in humans has rarely been examined and mainly focused on veterinary personnel, but according to the available data its prevalence is somewhere around 3.9–5.5% [6,40]. In our study we found a lower percentage (2.4%), but it should be taken into consideration that we screened owners and not veterinary staff. Prevalence of *S*. *pseudintermedius* in healthy dogs was 34.3% in this study. This figure is similar to that found by Fazakerley et al in the UK in 2009 (37.2%, at that time classified as *S*. *intermedius*) [41], but it is much lower compared to those reported more recently from Denmark (69%) [42] or Australia (85%) [43].

### 4.2. Antibiotic susceptibility

Tetracycline is one of the most frequently used antibiotic in livestock [44] hence the presence of *tet(K)* is very common in livestock associated and animal adapted strains [45,46]. The prevalence of *Dirofilaria immitis*, a helminth causing heartworm disease in carnivores has been rapidly increasing in Hungary since the early 2000’s [47,48]. A four week course of doxycycline therapy is part of the management of this disease in dogs [49] which could possibly lead to increased doxycycline use in small animal veterinary practice. This strengthens the possibility that our *S*. *aureus* isolates harbouring *tet(K)* had originated from animal source. We found that the generally animal adapted *S*. *pseudintermedius* strains showed significantly higher resistance to tetracycline than the predominantly human adapted *S*. *aureus* isolates (57% and 8% respectively, p = 0.0001).

60% of *S*. *aureus* showed elevated penicillin MICs whilst only 32% of *S*. *pseudintermedius* were resistant (p = 0.03). Possibly ß-lactamase production is in the background, as all isolates were susceptible to oxacillin and cefoxitin, furthermore, the *blaZ* gene was found in the *S*. *aureus* genomes. Despite the fact that penicillins are one of the most commonly used antibiotics in small animal medicine [50–53], we found low level of penicillin resistance in the animal derived *S*. *pseudintermedius*. These results are somewhat surprising considering the fact that usually higher resistance is documented in the literature. According to the annual nationwide reports of the National Health Care Institute of Hungary [54], the penicillin resistance of *S*. *aureus* isolates from outpatients is around 86–90% (although decreasing yearly) and ~90% resistance was measured in our previous human carriage studies as well [37].

Two of the *S*. *aureus* strains contained *tet(K)* gene resulting in tetracycline resistance. In the Q47-85 pair the WGS showed amino acid replacements in GrlA which could manifest in fluoroquinolone resistance [24], but our isolates showed 100% ciprofloxacin susceptibility.

Other antibiotic resistance coding genes were found but did not result in elevated MIC values (Table 3). Hypothetically these genes could cause resistance if upregulated or promoted under specific circumstances, such as direct antibiotic pressure during treatment.

Both sequenced *S*. *pseudintermedius* isolates were sensitive to all the examined antibiotics and no resistance genes were detected with WGS.

### 4.3. Clonal relatedness

The PFGE pulsotypes proved to be rather diverse (especially for *S*. *pseudintermedius*), which is typical in case of asymptomatic carriers, but one dominant clone was identified in case of *S*. *aureus* at >90% similarity level, which was represented by the two ST15 isolates (Fig 2). Furthermore, similarities were observed even between strains from different geographical locations. These results can possibly indicate that there are major types circulating in the community, nonetheless the number of isolates is limited to draw firm consequences. Owners and their dogs did share similar PFGE patterns indicating transmission via direct transfer from animals to humans or vice versa.

Clonal complexes CC15 and CC45 found in our study are primarily associated with isolates from humans [55]. However, according to the MLST database, in the last few years ST15 samples were identified from animal carriers as well.

Both members of the Q37 (human) and Q38 (canine) *S*. *aureus* sample pair was typed as ST15-t084. Surprisingly it seems to be frequently isolated from animals. ST15-t084 was detected during an *S*. *aureus* carriage survey from cows in Iran [56] and in several wildlife studies from different animals like naked mole rat, banded mongoose, Egyptian fruit bat, wild boar in Germany, Denmark and Spain [57]. The closely related ST15-t085 type was detected from an environmental sample in a veterinary hospital. [3]. The t084 was the most frequently isolated spa type from blood stream infections (BSI) in a Norwegian retrospective study in the early 2000’s [58].

The other *S*. *aureus* sample pair (Q47-Q85) belonged to the ST45 clonal type. These samples belonged to different but related spa types: t630 and t671. Presumably, these minor genetic variations evolved in the two different hosts, after interspecies transmission. ST45 has been for instance the predominant type among Belgian MRSA isolates in 2003 [59], and it was present also in Hungarian MRSAs—with low prevalence—in the early 2000s [60], but it was not found among recent Hungarian BSI MRSA strains [61]. The so-called USA600 MRSA also belongs to this clonal complex. According to the MLST database, members of ST45 were previously isolated from human carriers in Germany, Philippines and from an asymptomatic dog in the USA [28]. ST45 was also identified earlier in our carriage studies (represented by both MRSAs and MSSAs), from children and adults [62,63], veterinarians and dogs [unpublished], so it seems to be a common carried type in Hungary, being present for a long time. This assumption is further supported by the fact that Q47 and Q85 were isolated from two different geographical areas in Hungary.

### 4.4. Toxin genes

Our *S*. *pseudintermedius* isolates harboured exfoliative toxins genes (*siet*, *expB*), the expression of which can cause skin infection in dogs [64–66]. Furthermore, an ecthyma-like, painful, enlarging crusting lesion caused by a similar exfoliative toxin producing *S*. *pseudintermedius* strain in a husky dog owner has been documented previously [12], suggesting that hypothetically these isolates could be pathogenic to human hosts as well.

The *S*. *pseudintermedius* isolates (Q81-Q82) and one pair of the *S*. *aureus* strains (Q47-Q85) carried different kind of staphylococcal enterotoxins (SE). *S*. *aureus* food intoxications are well documented whereas the association of *S*. *pseudintermedius* with food poisoning is scarcely reported because it is mainly seen as a veterinary pathogen. However, as it was previously documented, both *S*. *pseudintermedius* and *S*. *intermedius* have occasionally been found in raw or processed food hence can be linked to human food-related outbreaks [15]. In this study, the *sec-int* gene could be detected in the *S*. *pseudintermedius* strains.

In both *S*. *aureus* isolates (Q47-Q85) we detected the enterotoxin gene cluster (*egc*) which contains the following toxin genes: *seg*, *sei*, *sem*, *sen*, *seo*, *seu*. This cluster is frequently recovered from dog and livestock samples [67–70]. Among the *S*. *aureus* isolates, Q47 contained the most types of enterotoxins and this human strain possessed the only classical SE (a-e): the *sec* subtype 2. Previously the *sec* gene was commonly found in bovine samples and human MRSA isolates, but it appeared in samples of dog origin as well [67,69–75]. In Q47 *sel* was also identified which has already been described from raw meat samples [70].

## 5. Conclusions

In this study we publish the first data documenting asymptomatic carriage of staphylococci among dogs and owners in Hungary. Based on the carriage rate and antibiotic susceptibility results—high tetraycline resistance—we can assume that the *S*.*pseudintermedius* strains are dominantly animal adapted. On the other hand, the majority of *S*. *aureus* isolates came from human sources and went through minor genetic changes during host switch and adaption.

The co-carried *S*. *aureus* samples Q37-Q38 both belonged to the ST15-t084 type. Although ST15 is a widely distributed and carried clonal complex, the genetic identity of Q37-Q38 is also mirrored in their virulome and resistome.

Minor differences were found in the Q47-Q85 samples. As these came from different geographical locations, they could have acquired different genes and mutations. We also found *S*. *pseudintermedius* in an owner and her dog (Q81-Q82) with identical genome which supports the literature data that humans can be colonised by these bacteria asymptomatically. These findings support the theory that dogs can act as reservoirs of staphylococci and can be the source of human infections. It is also likely that some major clonal types are circulating in the community in Hungary.

The strains possessed biofilm producing genes. In hospital settings they could be responsible for serious, medical device associated infections. Some of the isolates carried enterotoxins hence could be associated with food poisoning cases if they would contaminate food. *S*. *pseudintermedius* also contained several exfoliative toxins which could lead to skin infections.

Although our survey has its own limitations, it certainly draws attention to the fact that hospitalized patients and risk populations could develop infections originating from the bacterial flora carried by their own pets.

## Supporting information

S1 Raw images The original *S*. *aureus* and *S*. *pseudintermedius* PFGE gel pictures. (PDF)
