# Supplementary material for: Whole genome sequencing of coagulase positive staphylococci from a dog-and-owner screening survey
Source: PLoS One. 2021 Jan 11;16(1):e0245351. doi: 10.1371/journal.pone.0245351 (PMC7799803; doi:10.1371/journal.pone.0245351)

Figs 3 and 4 were generated from these original PFGE pictures.

The only modifications that occurred was cropping of images to exclude the non-gel background or adjusting contrast of the whole image to make ladders and or bands more visible.

Ladder: lamda (λ) ladder (CHEF DNA size standard; catalog no. 170-3635; Bio-Rad)

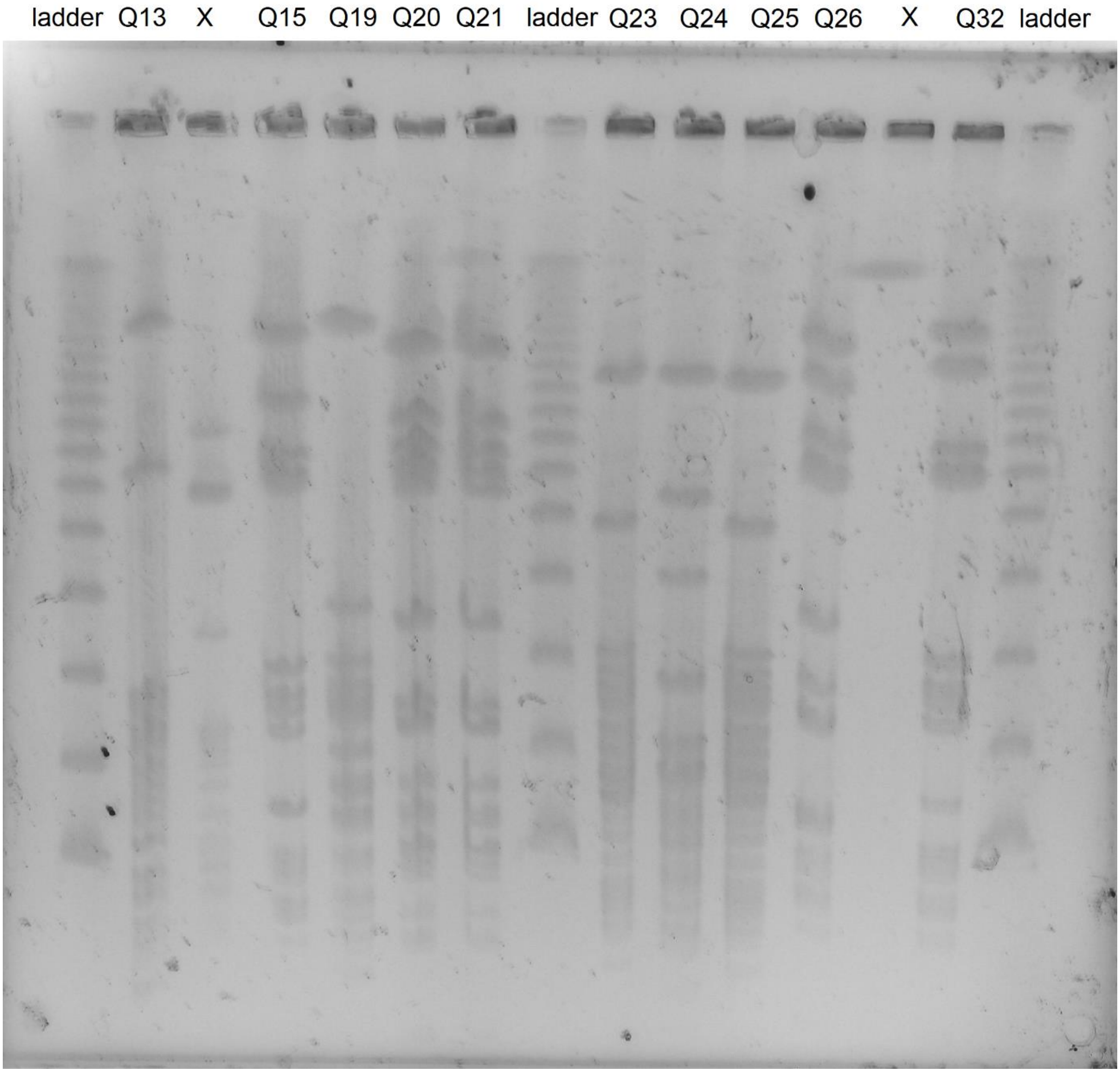

Figs 3 and 4 were generated from these original PFGE pictures.

The only modifications that occurred was cropping of images to exclude the non-gel background or adjusting contrast of the whole image to make ladders and or bands more visible.

Ladder: lamda (λ) ladder (CHEF DNA size standard; catalog no. 170-3635; Bio-Rad)

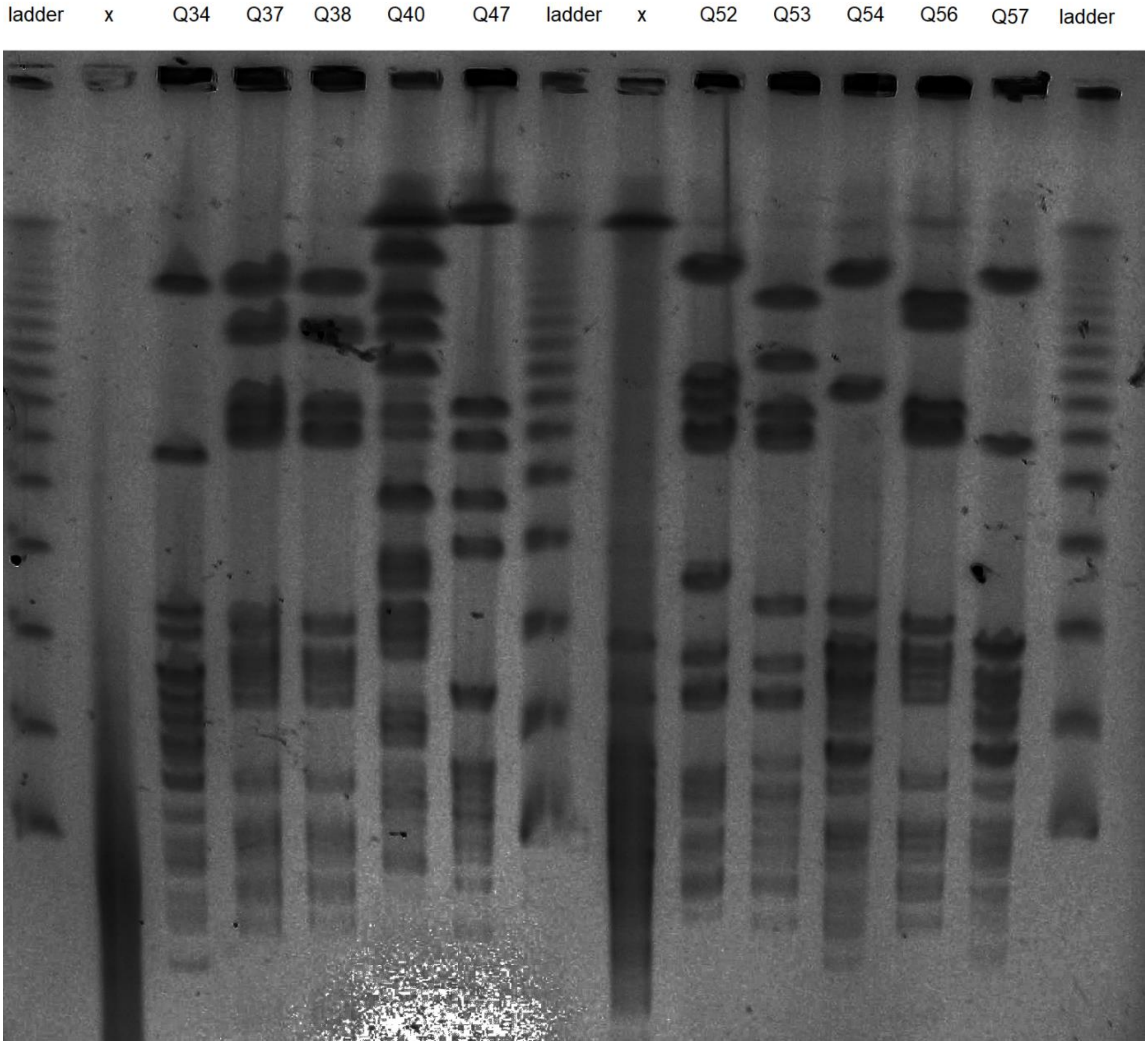

Figs 3 and 4 were generated from these original PFGE pictures.

The only modifications that occurred was cropping of images to exclude the non-gel background or adjusting contrast of the whole image to make ladders and or bands more visible.

Ladder: lamda (λ) ladder (CHEF DNA size standard; catalog no. 170-3635; Bio-Rad)

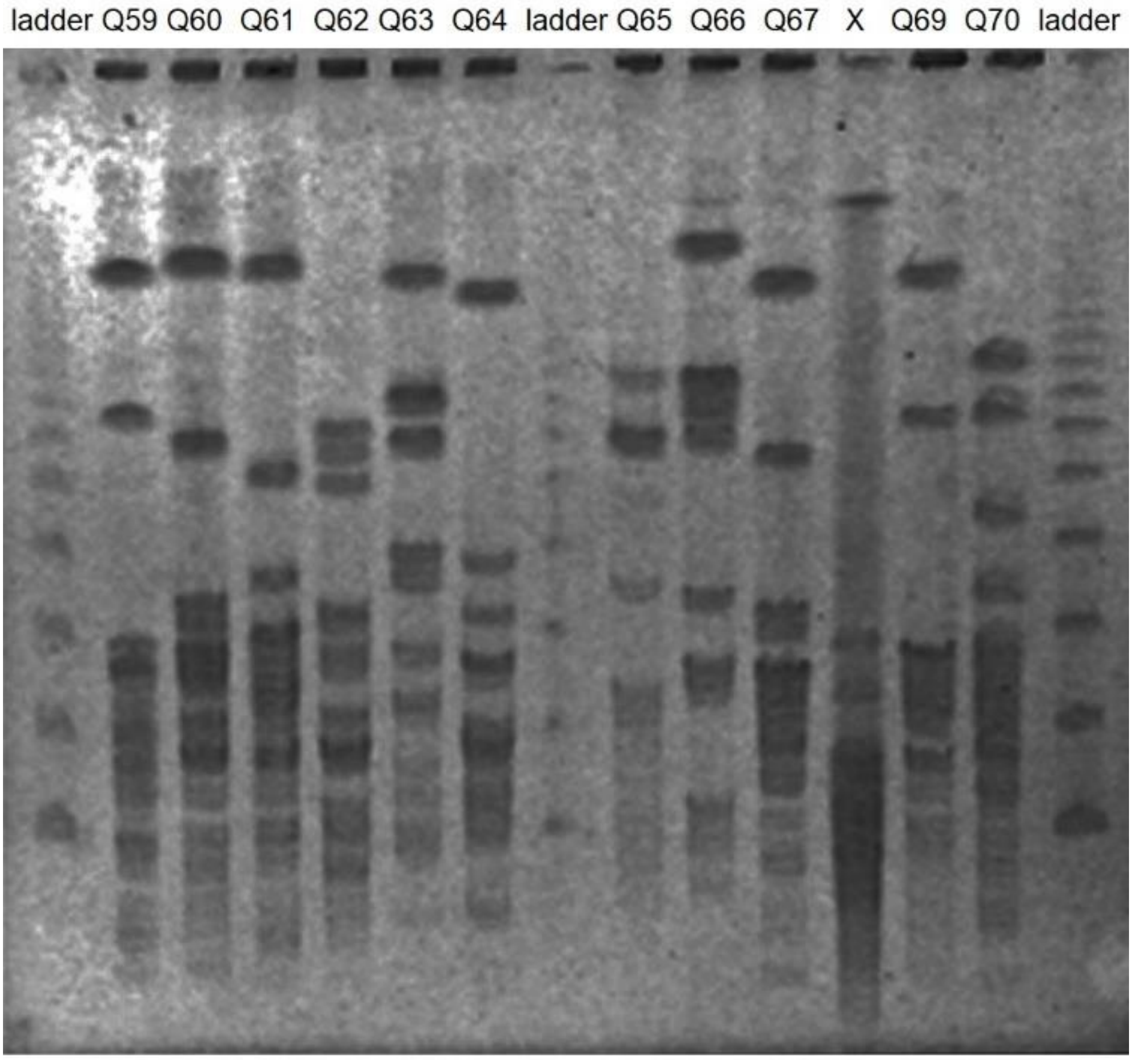

Figs 3 and 4 were generated from these original PFGE pictures.

The only modifications that occurred was cropping of images to exclude the non-gel background or adjusting contrast of the whole image to make ladders and or bands more visible.

Ladder: lamda (λ) ladder (CHEF DNA size standard; catalog no. 170-3635; Bio-Rad)

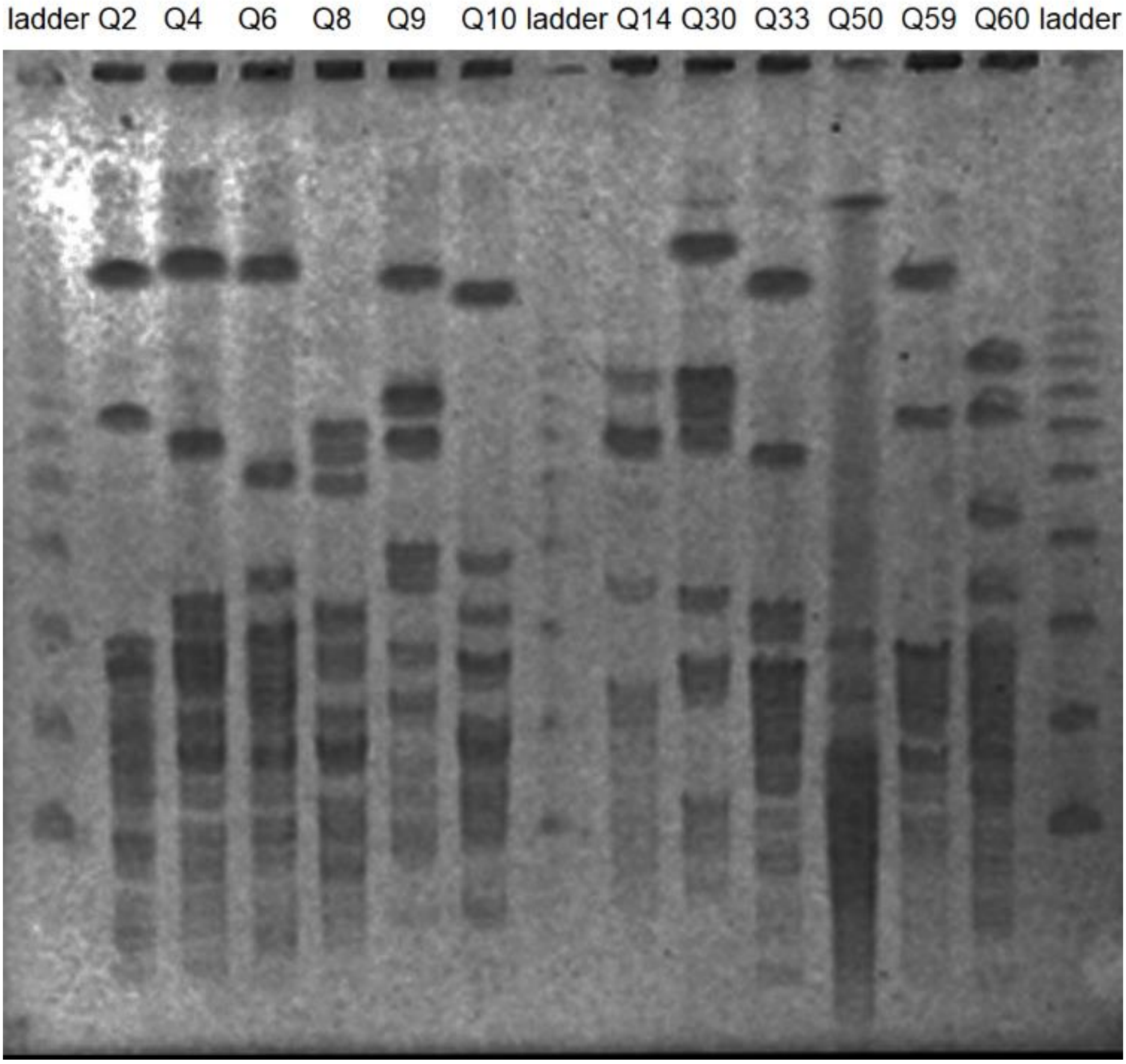

Figs 3 and 4 were generated from these original PFGE pictures.

The only modifications that occurred was cropping of images to exclude the non-gel background or adjusting contrast of the whole image to make ladders and or bands more visible.

Ladder: lamda (λ) ladder (CHEF DNA size standard; catalog no. 170-3635; Bio-Rad)

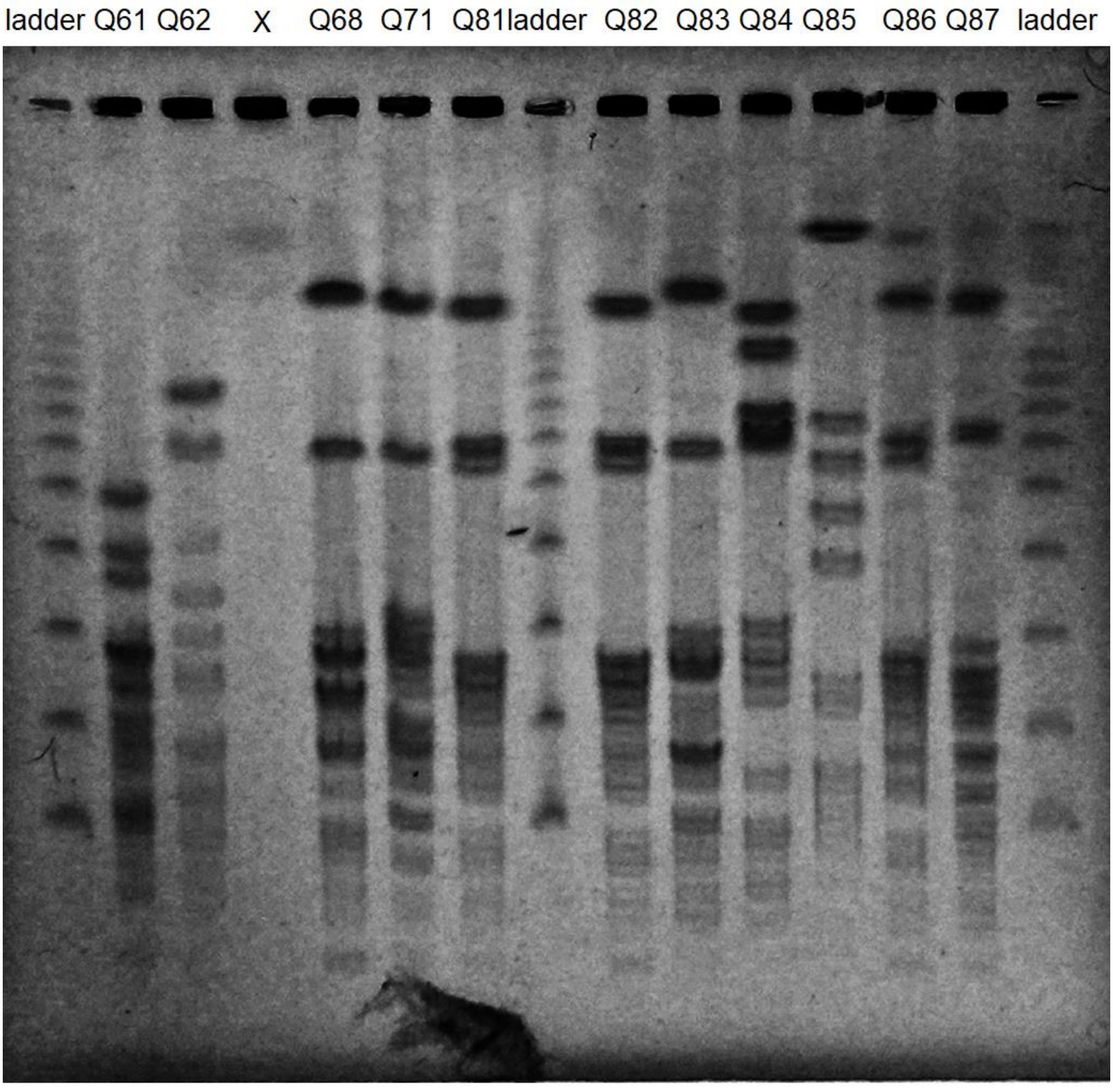

Figs 3 and 4 were generated from these original PFGE pictures.

The only modifications that occurred was cropping of images to exclude the non-gel background or adjusting contrast of the whole image to make ladders and or bands more visible.

Ladder: lamda (λ) ladder (CHEF DNA size standard; catalog no. 170-3635; Bio-Rad)

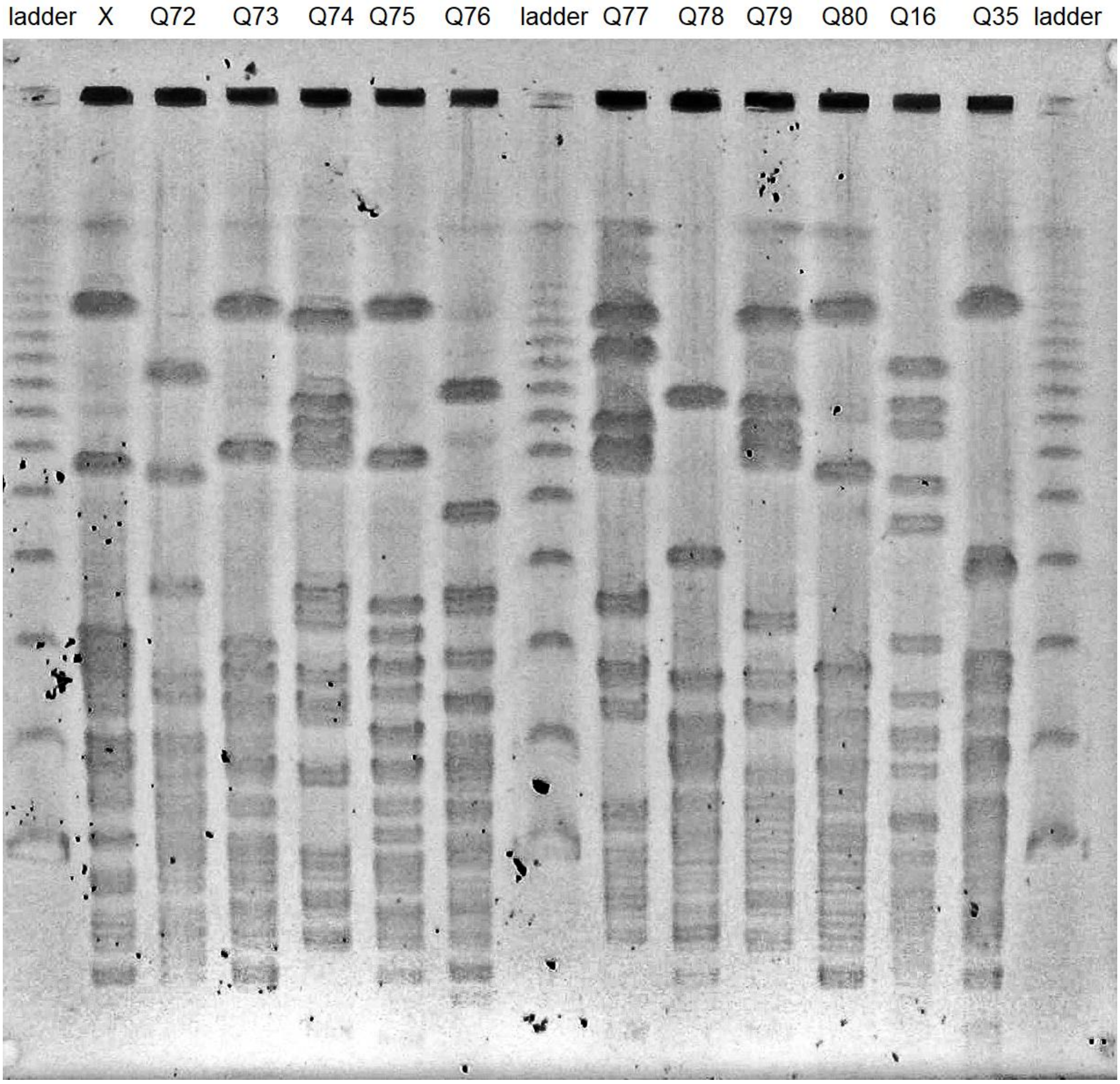

Supplement: S1 Raw images — (PDF) [file pone.0245351.s001.pdf]
